# 

**Published:** 1842-01-29

**Authors:** 


*The Medical Examiner* is published every Saturday by *J. C. Turnpenny*, N. E. corner of Spruce and Tenth streets. Terms—*three dollars* a year for one copy, or *five dollars* for two copies sent to the same post office, in all cases in advance. Postage the same as on newspapers. Letters to be addressed,—post-paid,—to the Editors of the Medical Examiner, Philadelphia.

*Reynell Coates, M. D.*, Acting Editor.

*J. B. Biddle, M. D.*, and *W. W. Gerhard, M. D.*, Co-editors.

*J. B. Biddle, M. D.* Proprietor.

